# SUBJECTIVE COGNITIVE DECLINE IN OLDER ADULTS: THE ROLE OF Α-SYNUCLEIN AND AMYLOID PATHOLOGIES

**DOI:** 10.1093/geroni/igae098.2279

**Published:** 2024-12-31

**Authors:** Kelsey Thomas, Katherine Bangen, Lindsay Rotblatt, Duygu Tosun, Douglas Galasko

**Affiliations:** University of California San Diego, La Jolla, California, United States; University of California, San Diego, La Jolla, California, United States; VA San Diego Healthcare System, San Diego, California, United States; University of California San Francisco, San Fransisco, California, United States; University of California, San Diego, La Jolla, California, United States

## Abstract

Some studies show subjective cognitive decline (SCD) to be an early marker associated with Alzheimer’s pathology. However, until recently, it has been impossible to measure Lewy body pathology in vivo, so its association with SCD is unknown. Participants without dementia (451 CN; 476 MCI) from the Alzheimer’s Disease Neuroimaging initiative were classified as positive or negative for amyloid (A+ or A-) using cerebrospinal fluid (CSF) Aβ42 and for α-synuclein (α-syn+ or α-syn-) using the CSF Seed Amplification Assay (441 A-/α-syn-, 80 A-/α-syn+, 331 A+/α-syn-, and 75 A+/α-syn+). Both participant- and study partner-reported SCD were measured using the Everyday Cognition (ECog) total and domain scores. Per self-report, after adjusting for age, education, sex/gender, race/ethnicity, APOE ε4+, and cognitive group, the A+/α-syn+ group had the greatest SCD relative to the other three pathology groups. The A-/α-syn+ group did not differ from the A-/α-syn- group on any ECog scores. None of these associations were moderated by cognitive status. Per study partner report, a similar pattern of results was observed, but the A+/α-syn- and A+/α-syn+ groups only significantly differed on the planning subscale despite A+/α-syn+ consistently having higher SCD. These results were moderated by cognitive group for the ECog total, memory, language, visuospatial, and divided attention scores such that associations were largely driven by the MCI group. In older adults without dementia, α-syn+ alone did not contribute to greater SCD; however, A+/α-syn+ had the greatest SCD as rated by both participant and study partner, highlighting the importance of measuring co-pathologies to understand SCD etiologies.

